# 

**Published:** 1863-05

**Authors:** 


### CHICAGO MEDICAL JOURNAL.

**Terms, $2.00 per Annum.**

### CONTENTS OF THE MAY NUMBER.

PAGE.

SELECTED.

Extract from a Letter to the Surgeon General of Massachusetts. From J. F. Gallupe, M D., Surg. 17th Mass. Vols ........ 193
Nature and Causes of Diphtheria ........ 195
Painless Parturition ........ 199
On the Employment of Mercury and the Iodide of Potassium in the Treatment of Syphilis. By Dr. Junien-Lavillauroy ........ 201
Pultaceous Exudations and False Membranes ........ 203
Bromide of Ammonium ........ 204
Researches on the Influence of Culinary Salt and Coffee on the Metamorphosis of Tissue ........ 204
Forces of the Living Organism ........ 205
Treatment of Joint Diseases ........ 209
Scarlatina and the Puerperal State ........ 210
Physical Disease in Insanity ........ 211

ORIGINAL COMMUNICATIONS.

Resections—Military Surgery. By Mord. Brooks, Asst. Surg. 82d Reg. Ind. Vols ........ 212
EDITORIAL AND MISCELLANEOUS ........ 414

